# Reactive vaccination as control strategy for an outbreak of invasive meningococcal disease caused by *Neisseria meningitidis* C:P1.5-1,10-8:F3-6:ST-11(cc11), Bergamo province, Italy, December 2019 to January 2020

**DOI:** 10.2807/1560-7917.ES.2022.27.24.2100919

**Published:** 2022-06-16

**Authors:** Cecilia Fazio, Laura Daprai, Arianna Neri, Marcello Tirani, Paola Vacca, Milena Arghittu, Luigina Ambrosio, Danilo Cereda, Maria Gramegna, Annapina Palmieri, Anna Carannante, Maria Rosa Bertoli, Lucia Crottogini, Giorgio Gennati, Eugenia Quinz, Livia Trezzi, Andrea Ciammaruconi, Silvia Fillo, Antonella Fortunato, Giovanni Rezza, Florigio Lista, Paola Stefanelli

**Affiliations:** 1 Department of Infectious Diseases, Istituto Superiore di Sanità, Rome, Italy; 2 Unit of Microbiology, Fondazione IRCCS Ca' Granda Ospedale Maggiore Policlinico, Milan, Italy; 3 Directorate General for Health, Lombardy Region, Milan, Italy; 4 Health Protection Agency of Milan, Milan, Italy; 5 Laboratory of Microbiology, Fondazione IRCCS Ca' Granda-Ospedale Maggiore Policlinico, Milan, Italy; 6 Chief Medical Laboratory of Clinical Chemistry and Microbiology, ASST Melegnano and Martesana, Milan, Italy; 7 Department of Cardiovascular, Endocrine-metabolic Diseases and Aging, Istituto Superiore di Sanità, Rome, Italy; 8 Health Protection Agency of Bergamo, Bergamo, Italy; 9 Scientific Departement, Army Medical Center, Rome, Italy; 10 Directorate General of Health Prevention, Ministry of Health, Rome, Italy

**Keywords:** *Neisseria meningitidis*, clonal complex 11, invasive meningocococcal disease, Italy, outbreak, serogroup C meningococcal vaccination

## Abstract

In Italy, serogroup C meningococci of the clonal complex cc11 (MenC/cc11) have caused several outbreaks of invasive meningococcal disease (IMD) during the past 20 years. Between December 2019 and January 2020, an outbreak of six cases of IMD infected with MenC/cc11 was identified in a limited area in the northern part of Italy. All cases presented a severe clinical picture, and two of them were fatal. This report is focused on the microbiological and molecular analysis of meningococcal isolates with the aim to reconstruct the chain of transmission. It further presents the vaccination strategy adopted to control the outbreak. The phylogenetic evaluation demonstrated the close genetic proximity between the strain involved in this outbreak and a strain responsible for a larger epidemic that had occurred in 2015 and 2016 in the Tuscany Region. The rapid identification and characterisation of IMD cases and an extensive vaccination campaign contributed to the successful control of this outbreak caused by a hyperinvasive meningococcal strain.

## Background

The main pillars to manage an outbreak of meningococcal disease are rapid detection and whole genome sequencing to identify gene signature and possibly reconstruct the chain of transmission among cases, as well as public health interventions such as a targeted vaccination campaign.

Most cases of invasive meningococcal disease (IMD) are caused by serogroup B, C, W and Y meningococci [1]. However, serogroup C meningococci (MenC) are more commonly associated with outbreaks or epidemics [2]. Epidemiological analyses and molecular characterisations of meningococci have identified a few clonal complexes (cc) considered to be hyperinvasive. Among the main hyperinvasive cc, the cc11 [3] is frequently associated with serogroup C and W and, more rarely, with serogroups B and Y. Cases of IMD caused by MenC/cc11 are often associated with sepsis and with a high case fatality rate (CFR) and sequelae [4]. Several outbreaks and epidemics due to MenC/cc11 have been described worldwide since the mid-1980s [3,5]; since 2013, outbreaks have been reported also in European countries such as Belgium, France, Germany, Italy and the United Kingdom (UK) [6-9].

Italy is a low incidence country for IMD, with an average incidence around 0.31 cases per 100,000 inhabitants during the 3 years 2017 to 2019 [10]. Nevertheless, several outbreaks have been caused by cc11 strains during the past 15 years [11-13]. Notably, an outbreak of 62 cases in the Tuscany Region occurred in 2015 and 2016, with a CFR of ca 21% [9].

Sequencing of the entire genome of *N. meningitidis* is considered the most cost-effective typing method in routine meningococcal surveillance and in particular in outbreak investigations. Moreover, phylogenetic analysis permits to highlight similarity among the strains and the presence of specific clusters [2]. A targeted vaccination strategy is key to control an outbreak.

## Outbreak detection

**Figure 1:**
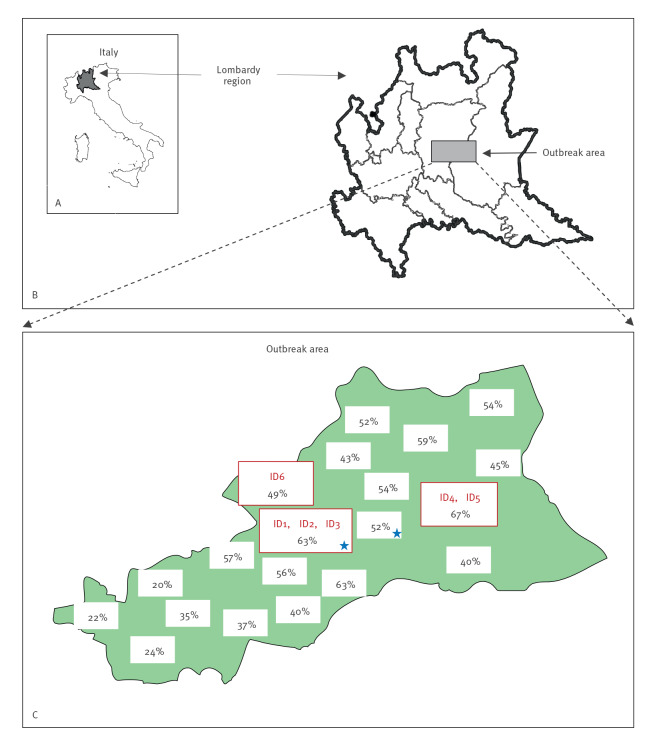
Geolocalisation of invasive meningococcal disease cases involved in the outbreak, Bergamo province, Italy, December 2019–January 2020 (n = 6)

Between 2 December 2019 and 29 January 2020, an outbreak of six IMD cases caused by MenC occurred in a limited area of the Bergamo province, Lombardy region, northern Italy (Figure 1). The temporal sequence of cases is described in the Table.

ID: case identification number.

Panel A shows a map of Italy with the Lombardy Region highlighted.

Panel B shows a map of the Lombardy Region with the outbreak area highlighted.

Panel C shows the six IMD cases (ID1–6) by site of isolation and the percentage of population coverage for MenC or MenACWY vaccines in the 20 villages of the outbreak area following the extraordinary vaccination campaign conducted after the fourth case.

Blue stars: places of residence and/or work of the first three cases.

**Table:** Epidemiological and clinical characteristics of MenC invasive meningococcal disease cases involved in the outbreak, Bergamo province, December 2019–January 2020 (n = 6)

ID	Day after the outbreak onset	Age group (years)	Clinical picture	Outcome
1	First day	15–24	Sepsis	Fatal
2	1 day after	15–24	Sepsis	Recovered
3	19 days after	25–49	Sepsis/meningitis	Recovered
4	31 days after	25–49	Sepsis	Fatal
5	39 days after	50–64	Sepsis/meningitis	Recovered
6	58 days after	≥ 65	Sepsis	Recovered

ID: case identification number.

Here we report the epidemiological and molecular analysis of the outbreak and its successful containment strategy.

## Methods

### Invasive meningococcal disease surveillance system

The IMD National Surveillance System (NSS) is coordinated by the national reference laboratory (NRL) of the Istituto Superiore di Sanità (ISS) with the support of the Italian Ministry of Health. The NRL receives bacterial isolates and/or clinical samples (blood and cerebrospinal fluid) from IMD cases, collected by the hospital laboratories, to perform serogroup identification/confirmation and molecular investigations. Meningococci isolated from the outbreak here described (five bacterial isolates and one clinical sample) were collected from hospital laboratories and sent to the NRL.

We used the outbreak definition by the ECDC: “*The occurrence of more cases than expected in a particular population, in a specific geographical area and over a specified period of time*” [14] and cases for this outbreak were defined as persons with confirmed IMD caused by MenC in the Bergamo province from December 2019 onwards.

### Microbiological and molecular analyses

For each meningococcal isolate, the serogroup was determined by slide agglutination with commercial antisera (Thermo Scientific, Waltham, United States (US)) or, for the clinical sample, by multiplex PCR [15]. For the bacterial isolates, susceptibility to cefotaxime, ceftriaxone, ciprofloxacin, meropenem, penicillin G and rifampicin was determined on Mueller–Hinton agar (Thermo Scientific, Waltham, US) supplemented with 5% of sheep blood by the minimum inhibitory concentration (MIC) test strip method (Liofilchem, Roseto degli Abruzzi, Italy). The breakpoints were those recommended by the European Committee on Antimicrobial Susceptibility Testing [16].

Chromosomal DNA was extracted using the QIAamp mini kit (Qiagen, Hilden, Germany) from an overnight culture or directly from the clinical sample (blood). Molecular typing of the bacterial isolates was done through whole genome sequencing. For the clinical sample, multilocus sequence typing (MLST), PorA and FetA typing were performed by Sanger sequencing, referring to the PubMLST.org database (http://pubmlst.org/neisseria) [17]. The MLST defined the sequence type (ST) and the cc. PorA and FetA types together contribute to the finetype. For each sample, we adopted the standard typing nomenclature [18] comprising serogroup: PorA type:FetA type:ST (cc). Moreover, the Neis0430 gene, coding for a cytoplasmic axial filament protein (*cafA*), was also sequenced to compare the Neis0430 allele in our cases with the one characteristic of the meningococcal strain responsible for the Tuscany outbreak (Neis0430 allele 398) [19].

### Whole genome sequencing

Cultivated isolates were analysed by whole genome sequencing. For each isolate, 1 ng of DNA was used to prepare the sequencing libraries following the Nextera XT DNA (Illumina) protocol (Document # 15031942 v05, May 2019). The Illumina MiSeq platform, with the reagent kit v3, 600 cycles, was used for the whole genome sequencing analysis. A first quality check of the raw sequence data was performed using FastQC [20]. Reads were trimmed using the software Sickle [21] to maintain a Q score > 25, and de novo assembly was carried out with the ABySS software version 1.5.2 (k parameter = 63) [22]. Contigs longer than 500 bp were selected using an ad hoc script and kept for further analysis. The final assembly ranged from 84 to 316 (median: 209) contigs per sample (N50: 10,999–59,092 bp; median: 19,790 bp), covering the ca 2.2 Mb of the *N. meningitidis* genome.

### Genome comparison

Genomes, uploaded to the PubMLST.org database (http://pubmlst.org/neisseria), were analysed and compared using the BIGSdb Genome Comparator through gene-by-gene analysis [23]. Through core genome MLST (cgMLST), a phylogenetic analysis was carried out [24]. Incomplete loci were automatically removed from the distance matrix calculation for the NeighbourNet graphs. Based on the distance matrices, a NeighbourNet network was generated by SplitsTree4 (version 4.13.1) [25].

## Results

### Invasive meningococcal disease surveillance data

During the year 2019, 189 IMD cases were reported in Italy, with an incidence of 0.31 cases per 100,000 inhabitants. Thirty-eight cases occurred in Lombardy Region (18 serogroup B, nine serogroup C, eight serogroup Y, one capsule null locus (*cnl*) strain and two non-determined), with an incidence of 0.38 cases per 100,000 inhabitants (ISS database https://w3.iss.it/site/mabi, last accessed: 30 April 2021), in line with what had been observed in the previous years (0.35 in 2018 and 0.32 in 2017). However, 19 IMD cases occurred in Lombardy (10 serogroup B, seven serogroup C and two serogroup Y) in December 2019 and January 2020 vs only four cases during the same period of the previous year (two serogroup B, one serogroup C and one serogroup Y) (https://w3.iss.it/site/mabi/, last accessed: 30 April 2021). Of the seven MenC cases, six (four female and two male cases) occurred in a limited geographical area of the Bergamo province. The clinical picture was characterised by a rapidly evolving sepsis (n = 4) or meningitis and sepsis (n = 2). The median age was 40 years (range: 15–70 years). Two of the six cases were fatal (Table). None of the patients had been vaccinated against MenC.

### Microbiological analyses

The following samples were analysed by the NRL: five meningococcal isolates (ID 1, 2, 4, 5, 6) and one clinical sample (blood; ID 3). Infection with serogroup C was confirmed through serological tests and/or molecular analysis.

All five meningococcal isolates were found susceptible to cefotaxime, ceftriaxone, ciprofloxacin, meropenem, penicillin G and rifampicin. All isolates showed MIC values to penicillin G ranging from 0.125 to 0.25 mg/L.

### Molecular analyses

The molecular analyses showed that the six meningococci belonged to ST-11/cc11 and shared the identical finetype P1.5–1,10–8:F3–6. The 398 Neis0430 allele was found in all of them.

### Genome comparison

For all isolates (except for the ID 3 case), we analysed the genomes for a high-resolution comparison using the gene-by-gene approach of cgMLST. A total of 1,581 of the 1,605 core genome loci were included in the cgMLST analysis (we excluded 24 loci that were missing in all): of those, 1,552 core genome loci were identical in all the isolates, with a mean distance of 14 loci with allelic differences (data not shown).

We carried out an additional cgMLST comparison of the five outbreak genomes and representative genomes of meningococci with identical genotypic designation: 13 genomes from Italian sporadic cases from the period 2019 to 2020, four genomes from an outbreak on a cruise ship sailing along the Italian coast in 2012 [12], 11 genomes from an outbreak in Tuscany in 2015 and 2016 [9,19] and five genomes from an outbreak on Sardinia in 2018 caused by a strain with switched capsule B:P1.5–1,10–8:F3–6:ST-11(cc11) [13]. Among the genomes obtained from sporadic cases, 10 presented the Neis0430 allele 398 and three the allele 6.

A total of 1,597 of the 1,605 core genome loci were included in the cgMLST analysis (we excluded eight loci that were missing in all). A total of 1,039 core genome loci were identical in all the isolates, with a mean distance of 73 loci with allelic differences.

**Figure 2:**
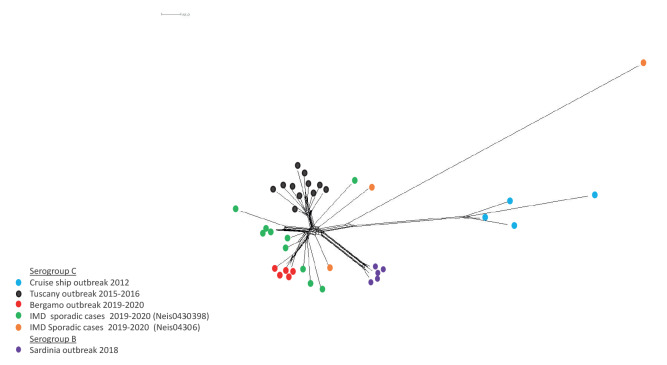
NeighbourNet phylogenetic network based on a comparison of 1,597 core genome loci (cgMLST) of meningococcal genomes C:P1.5–1,10–8:F3–6:ST-11(cc11) (n = 33) and B:P1.5–1,10–8:F3–6:ST-11(cc11) (n = 5) obtained from invasive meningococcal disease cases, Italy, 2012–2020

In the NeighbourNet network (Figure 2), reconstructed on the estimated allelic distances, we identified several genomes that were more closely related. In particular, genomes belonging to the same outbreak showed high proximity.

IMD: invasive meningococcal disease.

Sequences relate to the following outbreaks: Outbreak in a cruise ship sailing along the Italian coast of Tuscany, 2012 (n = 4) [12]; outbreak in Tuscany, 2015–2016 (n = 11) [9,19]; outbreak in Sardinia, 2018 (n = 5) [13]; outbreak in Bergamo province, 2019–2020 (n = 5); IMD sporadic cases 2019–20 Neis0430 allele 398 (n = 10); IMD sporadic cases 2019–2020 Neis0430 allele 6 (n = 3). The scale bar indicates the number of differences among the compared loci.

The five genomes isolated in the outbreak in Bergamo clustered together. They were in proximity of MenC:cc11 genomes deriving from representative samples of the outbreak in Tuscany and from more recent (2019–2020) sporadic cases, all characterised by the Neis0430 allele 398. Genomes belonging to the outbreak on Sardinia and the outbreak on the cruise ship, as well as three sporadic cases, all presenting the Neis0430 allele 6, were positioned further away from the genomes from Bergamo.

### Outbreak control measures

Epidemiological investigations, contact tracing and other public health measures such as chemoprophylaxis of close contacts were conducted by the regional Agency for the Protection of Health (Agenzia di Tutela della Salute), together with the local Agency for Territorial Social-Health (Azienda Socio Sanitaria Territoriale), the Directorate General Welfare, and the Organisational Unit Prevention. Vaccination strategies were designed by local health authorities in collaboration with the ISS and the Italian Ministry of Health.

After the first two cases, chemoprophylaxis with rifampicin (600 mg every 12 h for 2 days) and vaccination with a conjugate vaccine against serogroup C (MenC) or a quadrivalent conjugate vaccine against serogroups A, C, W and Y (MenACWY) were administrated to all close contacts of the six IMD cases. After identification of the third case, because it was determined by the same meningococcal serogroup and the geographical area affected by the outbreak was small, vaccination was extended to the overall population up to 50 years in the towns where the cases resided or worked (Figure 1, blue stars). After the fourth case, the vaccination campaign was extended to a wider geographical area, comprising places visited by the cases, contacts' places of residence and some villages geographically adjacent (Figure 1, green shading) and including the population up to 60 years of age.

Finally, of 67,571 inhabitants representing the entire population of the outbreak area, 28,070 were vaccinated with MenC or MenACWY within 9 weeks, (from 24 December 2019 to the first week of February 2020). After this prompt and extensive reactive vaccination campaign, no further MenC cases were notified in the outbreak area from February 2020 until the end of the year.

## Discussion

We identified an outbreak caused by a highly transmissible and hypervirulent MenC/cc11 strain. The same strain was has caused several outbreaks worldwide [2,3,5-8].

Outbreaks involving the C:P1.5–1,10–8:F3–6:ST-11(cc11) strain had already been identified in previous outbreaks in Italy [9,11-13]. In fact, the phylogenetic analysis highlighted the close genetic proximity between the strain responsible for the outbreak in Bergamo and that of a larger outbreak in Tuscany in 2015 and 2016 [9,19]. Both outbreaks were characterised by cases with severe disease and a CFR of more than 20%. The genetic relationship between the strains from Bergamo and Tuscany is also supported by the presence of Neis0430 allele 398. Of note, the C:P1.5–1,10–8:F3–6:ST-11(cc11) isolates with Neis0430 allele 398 responsible for sporadic cases throughout the country in the same period showed a genetic proximity with the genomes from Bergamo in the phylogenetic analysis. These cases were characterised by a high CFR (50%, data not shown).

In this outbreak, all patients except one were older than the recommended vaccination target age groups in Italy. The national immunisation plan for the period 2017 to 2019 recommended the use of MenC vaccine during the second year of life and the MenACWY vaccine from 12 to 18 years of age [26].The immunisation campaign has provided the MenC vaccine for children up to 10 years of age, MenACWY for adolescents, and MenC or MenACWY for adults up to 60 years of age. 

## Conclusion

Meningococcal outbreaks caused by hypervirulent strains require rapid identification and response with specific public health measures. In parallel with a prompt post-exposure prophylaxis, also a reactive vaccination campaign proved necessary in order to contain the outbreak, preventing new cases and deaths. Rapid microbiological identification and characterisation of the outbreak strain is also important to plan a timely vaccination campaign.
